# Epstein-Barr virus mRNA vaccine synergizes with NK cells to enhance nasopharyngeal carcinoma eradication in humanized mice

**DOI:** 10.1016/j.omton.2025.200986

**Published:** 2025-04-24

**Authors:** Kun Huang, Xiao-jun Lin, Jing-chu Hu, Ting-ying Xia, Feng-ping Xu, Jian-dong Huang, Nan Zhou

**Affiliations:** 1 BGI Cell, Shenzhen 518083, China; 2 Key Laboratory of Quantitative Synthetic Biology, Shenzhen Institute of Synthetic Biology, Shenzhen Institutes of Advanced Technology, Chinese Academy of Sciences, Shenzhen 518055, China; 3 School of Biomedical Sciences, Li Ka Shing Faculty of Medicine, The University of Hong Kong, Pokfulam, Hong Kong SAR, China

**Keywords:** MT: Regular Issue, Epstein-Barr virus, nasopharyngeal carcinoma, epitopes, mRNA vaccine, natural killer cells, combined therapy, humanized mouse model

## Abstract

The close association between nasopharyngeal carcinoma (NPC) and Epstein-Barr virus (EBV) infection highlights the potential of therapeutic vaccination against viral antigens as an attractive immunotherapy for treating EBV^+^ NPC. Maximizing vaccine efficacy often requires selecting optimal T cell epitopes and incorporating co-treatment strategies. Here, we analyzed genomic mutations of 283 cancer-associated EBV strains and predicted epitopes with broad human leukocyte antigen (HLA) coverage from high-frequency nonsynonymous mutations. A polyepitope mRNA vaccine constructed from the predicted epitopes elicited antigen-specific T cell responses but showed suboptimal efficacy in tumor control in a PBMC-humanized mouse EBV^+^ NPC model. To enhance treatment efficacy, we developed an optimized system for expanding human natural killer (NK) cells with high purity and cytotoxicity as a co-treatment modality. Combined administration of mRNA vaccine and NK cells synergistically improved therapeutic efficacy by durably suppressing or eradicating NPC tumors in humanized mice. The concurrent treatment could improve the infiltration of both human T cells and NK cells into the tumor microenvironment and boost their effector functions. Our study suggests the combined therapeutic vaccination and NK cell therapy as a potential strategy for treating EBV^+^ NPC.

## Graphical abstract

**Figure:**
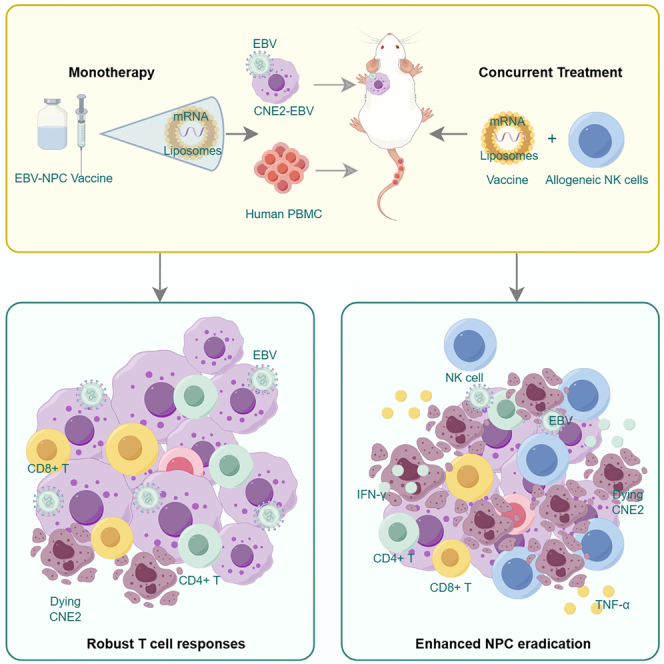

## Teaser

Combining an EBV-mutation-targeted mRNA vaccine with expanded allogeneic natural killer (NK) cells synergistically eradicated nasopharyngeal carcinoma in humanized mice by overcoming tumor immune evasion and enhancing both adaptive T cell and innate NK cell responses. This dual immunotherapy presents a promising strategy against EBV-driven malignancies.

## Introduction

Nasopharyngeal carcinoma (NPC) is the predominant malignancy of the nasopharynx, and its occurrence shows considerable geographic variation.^1^ Despite its low incidence rates in most regions of the world, NPC, particularly the nonkeratinizing undifferentiated subtype, is endemic to North Africa and Southeast Asia.^2^ Conventional standard treatments for NPC include surgery, radiotherapy, chemotherapy, and concurrent chemoradiotherapy, which are effective in patients with locoregional disease (85%–90% survival over 5 years).^3^^,^^4^^,^^5^ However, the undifferentiated NPC is highly invasive and likely to metastasize, which may lead to the failure of primary treatment.^6^^,^^7^ Currently, immunotherapies such as immune checkpoint inhibitors (ICIs) and adoptive cell therapy (ACT) are under active investigation for NPC treatment.^8^ They have demonstrated significant benefits in multiple clinical trials, offering new avenues to improve therapeutic outcomes in patients with advanced NPC.^9^

Most undifferentiated NPCs have an etiological association with Epstein-Barr virus (EBV) infection.^10^ EBV can be detected in precancerous lesions and is crucial for NPC oncogenesis by inducing genomic instability and exerting immunosuppression.^11^^,^^12^ These properties have made EBV an appealing candidate for targeted NPC therapy.^13^ To this end, therapeutic vaccination has emerged as a potential treatment option by evoking the individual’s T cell immunity against viral antigens and thereby EBV-infected cancer cells.^8^^,^^14^ To date, various vaccine forms have been developed, with a primary focus on dendritic cells pulsed with EBV antigens and recombinant viruses expressing EBV antigens.^15^^,^^16^^,^^17^^,^^18^^,^^19^^,^^20^^,^^21^^,^^22^^,^^23^ Other approaches based on cancer stem cell lysates,^24^ synthetic peptides,^25^ plasmid DNA,^26^ and mRNA^27^ were also assessed. Early clinical trials have demonstrated the safety and immunogenicity of therapeutic EBV vaccines, with only a subset of patients exhibiting robust T cell responses.^28^ The wild-type oncoproteins EBNA1, LMP1, and LMP2 from prototype EBV strains such as B95-8 are the most commonly attacked antigens. Of note, recent studies using whole-genome sequencing have identified numerous genomic variations in cancer-related EBV strains.^29^^,^^30^ Similar to how somatic mutations in cancer cells can generate neoantigens,^31^ the unidentified non-synonymous mutations in EBV genomes may encode immunogenic neo-epitopes for vaccine design, and this possibility has yet to be experimentally validated.

Tumor vaccines activate adaptive immunity by delivering tumor antigens to dendritic cells (DCs), inducing antigen-specific CD8^+^/CD4^+^ T cell responses and long-term memory.^32^ However, their effectiveness is limited by tumor heterogeneity, major histocompatibility complex class I (MHC class I) downregulation, and immunosuppressive tumor microenvironments (TMEs).^33^ Currently, cancer vaccines often require concurrent treatments to overcome immune evasion and maximize effectiveness in treating established solid tumors.^34^ The investigation of combining therapeutic vaccinations with other modalities is an active area of research in NPC treatment.^35^ Given the increasing recognition of the pivotal role of the innate immune response in cancer immunity, the involvement of innate immune cells holds promise for multilayered and durable tumor control.^36^ Natural killer (NK) cells represent a specialized effector cell population that is innately primed against nascent aberrant transformed cells and virus-infected cells.^37^ NK-cell-mediated cytotoxicity does not depend on human leukocyte antigen (HLA), which can eliminate cancer cells such as EBV^+^ NPC cells that are prone to evade T-cell-mediated immune surveillance due to HLA loss.^38^^,^^39^ Studies have shown that a higher NK cell proportion in the TME is associated with improved prognosis in EBV^+^ NPC.^40^ A case report documented a progressive regression of brain metastases in an advanced NPC patient after allogeneic NK cell transfer.^41^ Safety profile and partial clinical response were also observed in phase I study combining allogeneic NK cells with cetuximab for the treatment of recurrent NPC, demonstrating the translational potential of adoptive NK cell therapy.^42^ Based on these circumstances, tumor vaccines combined with allogeneic NK cells are anticipated to elicit two-pronged anti-tumor effects by activating both the tumor-specific adaptive immunity and MHC-unrestricted cytotoxicity. Furthermore, synergy between the two therapies can emerge as vaccines upregulate NK-activating ligands on tumors, while NK-cell-mediated tumor lysis releases additional antigens. This process enhances antigen cross-presentation and cytokine secretion (e.g., interferon gamma [IFN-γ]), thereby amplifying vaccine-induced adaptive immunity and promoting cytotoxic T lymphocyte (CTL) recruitment.^43^ This bidirectional interaction may bridge innate and adaptive immunity, facilitating rapid tumor clearance and sustained immune surveillance. To date, the potential synergistic effect of NK cell therapy in combination with therapeutic vaccination for treating EBV^+^ NPC remains unexplored.

In this study, we analyzed genomic mutations of cancer-associated EBV strains and predicted epitope candidates potentially covering a broad spectrum of HLA alleles from high-frequency non-synonymous mutations. In a humanized NPC mouse model, we show that a polyepitope mRNA vaccine constructed from predicted epitopes can elicit antigen-specific T cell immune responses against EBV^+^ NPC. Moreover, through co-treatment with *in vitro* expanded human NK cells exhibiting high purity and cytotoxicity, the concurrent treatment durably suppressed or even eradicated NPC tumors in humanized mice. Our preclinical results showcase the potential of combining therapeutic vaccination with adoptive NK cell therapy to enhance the treatment efficacy for EBV^+^ NPC.

## Results

### Prediction of EBV epitopes with broad HLA coverage from genomic mutations of cancer-associated EBV strains

**Figure 1:**
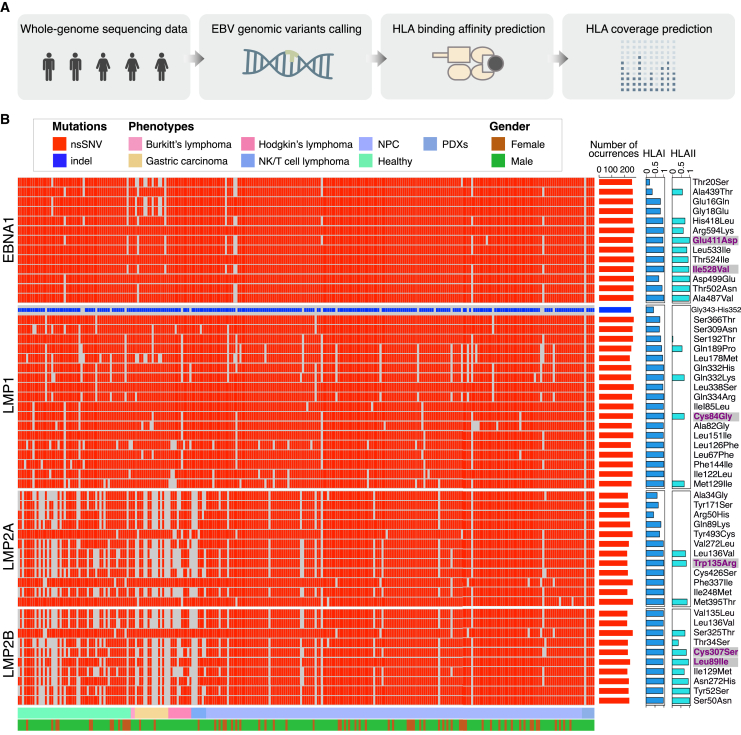
Prediction of immunogenic EBV epitopes with broad HLA coverage from genomic nsSNVs of cancer-related EBV strains (A) Schematic representation of the prediction pipeline. (B) Heatmap plot indicates the distribution of EBV genomic variations in different tumor types. Number of occurrences for each mutation in 283 sequenced strains, their HLA coverage, and exact amino acid substitution information are shown on the right. Mutations highlighted in purple are selected for vaccine construction due to their predicted strong HLA binding affinity and broad HLA coverage. Meta information, including tumor types and gender, is plotted at the bottom.

Genomic mutations present in tumors not only reflect the features of the tumor evolution but also serve as valuable targets for immunotherapies.^31^ Neoantigens that arise from tumor-specific nonsynonymous single-nucleotide variants (nsSNVs) or small-scale genome insertions and deletions (indels) have shown to be immunogenic for developing effective cancer vaccines.^44^^,^^45^ As viruses can develop a substantial number of genomic mutations during their evolution,^46^ we propose that particular nsSNVs or indels occurring in cancer-related EBV genomes may encode immunogenic epitopes suitable for constructing vaccines against EBV-associated malignancies. Therefore, we analyzed genomic variations of 283 cancer-associated EBV strains by using the B95-8 EBV strain of non-cancerous origin as a reference and predicted immunogenic epitopes encoded by those mutations (Figure 1A).

As a result, we discovered high-frequency nsSNVs in EBV oncogenes including EBNA1, LMP1, LMP2A, and LMP2B. We also confirmed a high-frequency indel in LMP1, featuring a deletion of 10 amino acids ranging from position 343 to 352. The elicitation of immune responses to a given epitope is typically restricted to individuals who possess an HLA molecule capable of binding and presenting that specific epitope. To design a T cell epitope-based EBV vaccine intended to be effective across diverse populations, we further predicted immunogenic EBV epitopes with broad HLA coverage from those mutations. To do so, we subjected variant peptides encoded by the nsSNVs to binding affinity prediction across diverse classes of HLA alleles, which have been widely used in antigen-MHC binding affinity prediction with high accuracy, for example, the neoantigen prediction from tumor patients.^47^ The HLA coverage for each mutation was calculated and shown in the right two columns of Figure 1B. Finally, a total of six epitopes with predicted broad population coverage for both HLAI and HLAII alleles were chosen for following mRNA vaccine construction (Table 1). Of the six mutations selected, five are located in previously identified T cell epitopes in EBNA1 and LMP2.^27^ One mutation in LMP1, namely Cys84Gly, corresponds to an epitope that has not been reported.^48^^,^^49^

**Table 1:** List of predicted EBV epitopes from cancer-associated EBV strains

ID	Gene	Mutated sequence used for vaccination	Substitution	Corresponding B95-8 epitope	HLAI coverage	HLAII coverage
E1	EBNA1	RPPPGRRPFFHPVG**D**ADYFEYHQEGGPDGE	E411D	HPVGEADYFEY	0.94	0.99
E2	EBNA1	KTSLYNLRRGTALA**V**PQCRLTPLSRLPFGM	I528V	IPQCRLTPL	0.99	0.95
L1	LMP1	FIFRRDLLCPLGAL**G**ILLLMITLLLIALWN	C84G	N/A	0.99	0.69
L2	LMP2A	NPVCLPVIVAPYLF**R**LAAIAASCFTASVST	W135R	PYLFWLAAI	0.99	0.81
L3	LMP2B	EWGSGNRTYGPVFM**S**LGGLLTMVAGAVWLT	C307S	TYGPVFMCL	0.99	0.81
L4	LMP2B	ICLTWRIEDPPFNS**I**LFALLAAAGGLQGIY	L89I	IEDPPFNSL	0.96	0.93

### mRNA vaccine induces antigen-specific T cell response against EBV^+^ NPC in humanized mice

**Figure 2:**
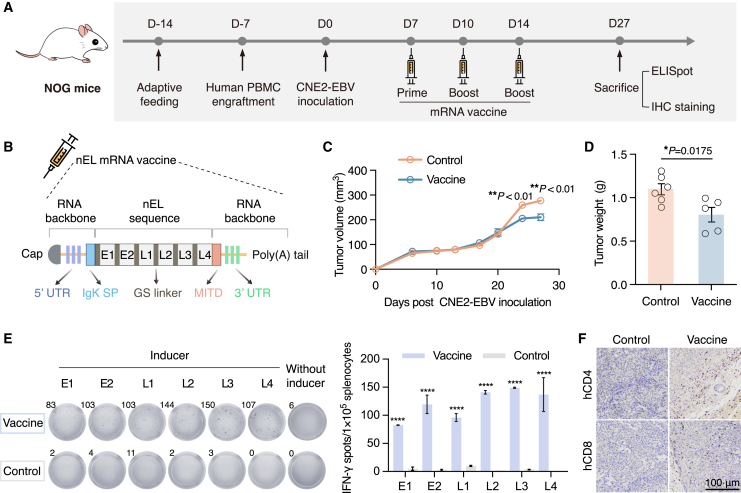
The nEL mRNA vaccine elicits antigen-specific T cell immune response against EBV^+^ NPC in humanized mice (A) Scheme of the animal experiment. Human PBMC-engrafted NOG mice were subcutaneously implanted with CNE2-EBV cells. One week after tumor implantation, mice were treated with nEL mRNA vaccine (vaccine) or liposomes (control) every 3 to 4 days (n = 5–6 per group). Spleen and tumor samples were collected for subsequent analysis at the end of the experiment. (B) A schematic diagram of the polyepitope nEL mRNA molecule. (C) Tumor growth curves of vaccinated and control mice (ordinary two-way ANOVA; ns, no significance; ∗∗*p* < 0.01). (D) Average tumor weight of vaccinated and control mice (unpaired two-tailed t test, ∗*p* < 0.05). (E) Post-vaccination response to the respective epitope for vaccinated and control mice. Representative images of splenic IFN-γ ELISpot assay (left) and the quantification of IFN-γ positive splenocytes (right, unpaired two-tailed t test, ∗∗∗∗*p* < 0.0001). (F) IHC staining of human CD4 and CD8 in tumor sections; scale bar, 100 μm. All data represented as means ± SEM.

The mouse model transplanted with human PBMCs can reconstitute human cellular immunity, therefore providing a useful tool to evaluate cancer vaccines and immunotherapies.^50^ To assess the immunogenicity as well as therapeutic potential of the human HLA-restricted epitopes predicted earlier, we synthesized a polyepitope EBV mRNA vaccine and tested its efficacy in a humanized mouse EBV^+^ NPC model. This model was established by inoculation of undifferentiated CNE2-EBV NPC cells in PBMC-humanized mice (Figures 2A and S1A). Graft-versus-host disease (GvHD) response is strictly monitored during the entire experimental window. None of the animals manifested weight loss exceeding 10% body mass or abnormal feeding behavior during the observation period post-humanization (Figures S1B and S1C). The synthetic mRNA vaccine, termed as nEL vaccine, consists of RNA molecules that encode all six epitopes connected by non-immunogenic glycine/serine linkers (Figures 2B and S2A). When transfected in HEK-293T cells, the mRNA expression product nEL could be successfully detected in the culture medium and cell lysate (Figure S2B). The *in vivo* transfection and expression capacity of the designed mRNA was also demonstrated by the live imaging of the mouse with intramuscularly injected EGFP encoding mRNA complexed with liposomes (Figure S2C).

In EBV^+^ NPC-bearing mice repeatedly vaccinated with the nEL vaccine, significant tumor growth inhibition effects were observed at days 24 and 27 (Figures 2C and S2D). Consistently, the weight of the tumor tissue harvested from the vaccine group was significantly less than that of the control group at the end of the experiment (Figure 2D). To further investigate whether vaccination could evoke antigen-specific T cell response, we tested splenocytes collected from both groups for their reactivity against the six epitopes by IFN-γ ELISpot. As a result, T cell responses were detected against 100% of the predicted epitopes in vaccinated mice, while such responses were almost absent in control mice (Figure 2E). Moreover, the infiltration of both human CD4^+^ and CD8^+^ T cells in tumor lesions of nEL vaccinated mice were significantly brisker in contrast to those observed in controls (Figure 2F). Collectively, the abovementioned data indicate that the nEL vaccine elicits robust T cell responses against each predicted epitope and reshapes the TME, albeit exhibiting limited efficacy in controlling tumor growth in a humanized mouse EBV^+^ NPC model.

### Production of high-quality human NK cells by an optimized expanding system

**Figure 3:**
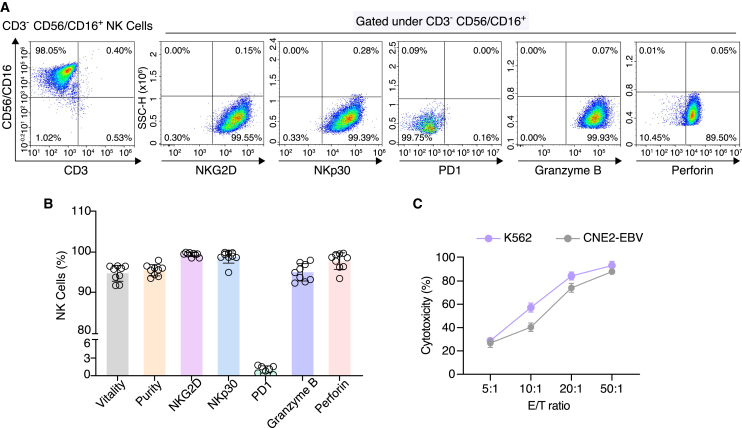
Phenotypic characterization of expanded NK cells from donor peripheral blood (A) Flow cytometric characterization of NKG2D, NKp30, PD1, granzyme B, and perforin expression in expanded NK cells. (B) Average vitality and purity of NK cells, percentages of expanded NK cells expressing NKG2D, NKp30, PD1, granzyme B, and perforin in nine individual experiments. (C) Cytotoxicity assay of expanded NK cells against the leukemic cells K562 and CNE2-EBV cells (*n* = 3 per group).

To ensure the consistent production of qualified human NK cells for the combination therapy with mRNA vaccine, we optimized various aspects of a previously described NK cell expansion protocol,^51^ including the starting population of PBMCs and the time window of proliferation and re-stimulation (see materials and methods for details). We examined the effector phenotype of our expanded NK cells in a greater detail by flow cytometry. As shown in Figure 3A, the expression of NK cell activation receptors NKG2D and NKp30 was observed in over 90% of expanded cells, while the expression of immune checkpoint PD1 was absent. Meanwhile, most NK cells (over 90%) could express granzyme B and perforin, indicating their cytotoxic phenotype (Figure 3B). The optimized NK expanding method was repeated in nine independent experiments, yielding an average of 3.3×10^9^ CD3^−^ CD56/CD16^+^ NK cells with above 90% viability and over 90% purity (Figure 3B). Notably, the expanded NK cells exhibited strong cytotoxicity (>60%) against both K562 and CNE2-EBV cells at 20:1 and 50:1 E/T ratios, suggesting that the expanded cells were functionally competent (Figure 3C). Taken together, our optimized NK cell expanding system enables the stable production of cytotoxic NK cells with high viability and purity.

### Combined therapeutic vaccination and NK cell therapy promotes EBV^+^ NPC eradication in humanized mice

**Figure 4:**
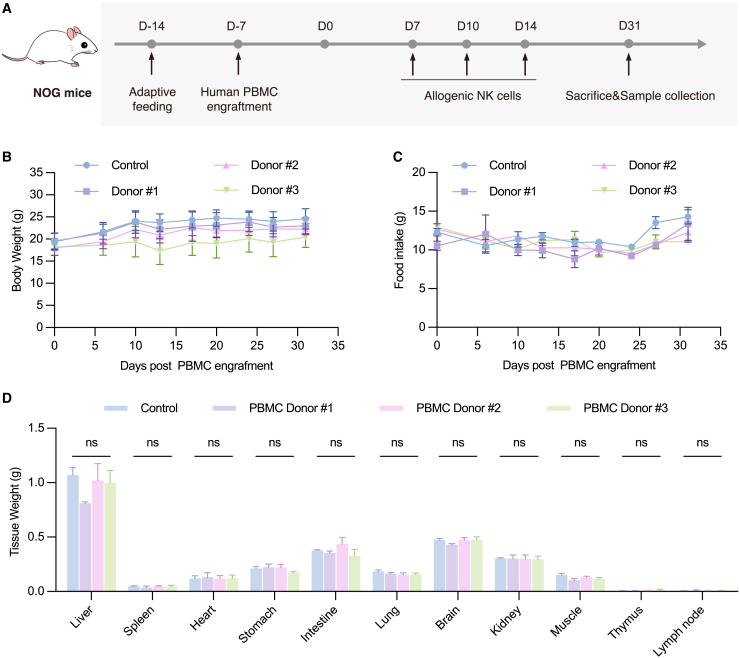
Assessment of systemic engraftment and physiological impacts in NOG mice following human PBMC transplantation and allogeneic NK cell administration (A) Scheme of the animal experiment. Two weeks after human PBMCs engraftment (from 3 different donors), NOG mice were intravenously injected with allogeneic NK cells or saline solution containing 1% human serum albumins (control) every 3 to 4 days (*n* = 3 per group). Tissue samples (liver, spleen, heart, stomach, intestine, lung, brain, kidney, muscle, thymus, and lymph node) were collected for subsequent analysis at the end of the experiment. (B) Body weight and (C) food intake changes of PBMC-humanized mice in the control and NK-injected groups (*n* = 3) at different time points. (D) Tissue weight analysis of major organs at the experimental endpoint (ordinary one-way ANOVA with Tukey’s multiple comparisons test; ns, no significance; *p* > 0.05). All data represented as means ± SEM.

Before testing the efficacy of the combined vaccination and adoptive NK cell therapy, we conducted a systematic evaluation of the safety profile and potential adverse effects associated with the adoptive transfer of human PBMCs and allogenic NK cells (Figure 4A), including twice-weekly monitoring of body weight, nutritional intake, cutaneous integrity, diarrheal incidence, comprehensive hematological profiling, and histopathological evaluation. As a result, none of the humanized mice manifested weight loss exceeding 10% body mass and abnormal food intake (Figures 4B and 4C). Gastrointestinal distress, erythema, kyphotic posture, pilomotor reflex abnormalities, or other clinical indicators of GvHD were also not detected during the observation period post-humanization and NK cell infusion. Comprehensive hematological profiling demonstrated successful engraftment and NK cell reconstitution, while other hematological indices remained within physiological ranges (Figure S3A), and serum analysis revealed no clinically relevant fluctuations in alanine aminotransferase (ALT), aspartate aminotransferase (AST), uric acid (UA), or urea concentrations, collectively confirming the absence of hepatorenal toxicity associated with the intervention (Figure S3B). Thirty-eight days post-transplantation, murine tissues and organs were systematically harvested for comparative mass analysis (Figure 4D). Comprehensive histological examination revealed preserved cytoarchitecture across all examined organs, with no evidence of pathological alterations (Figure S3C). Crucially, characteristic manifestations of GvHD, including apoptotic bodies and biliary plug formation as documented in human clinical cases,^52^ were conspicuously absent in the hepatic and pulmonary tissues of humanized murine subjects. These findings collectively demonstrate successful containment of GvHD pathogenesis in our experimental model, while simultaneously confirming the absence of significant toxicity associated with PBMC engraftment and NK cell infusion protocols.

**Figure 5:**
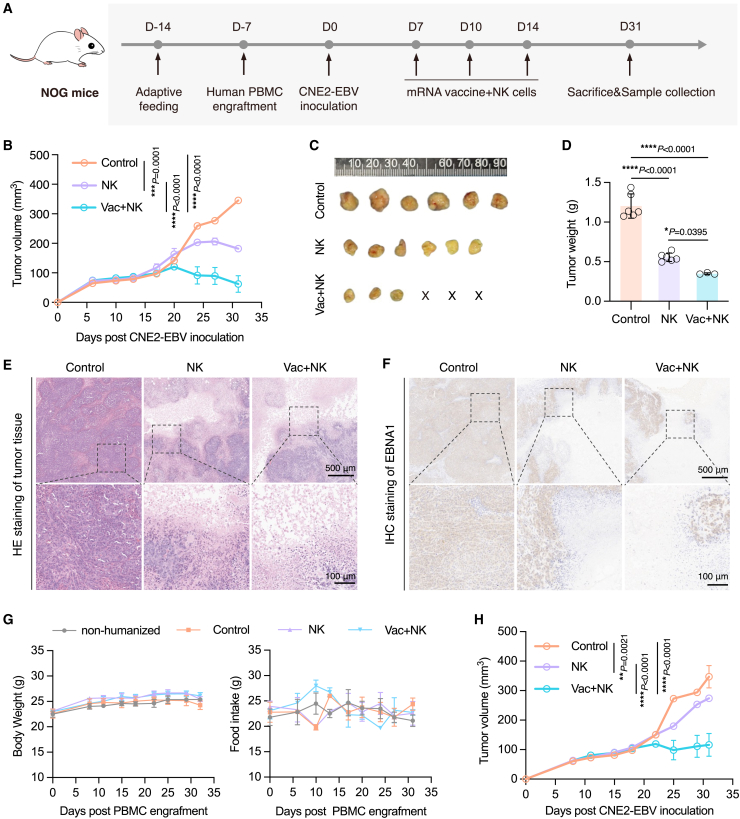
The nEL mRNA vaccine synergizes with NK cells to promote the eradication of EBV^+^ NPC in humanized mice (A) Scheme of the animal experiment. Human PBMC-engrafted NOG mice were implanted subcutaneously with CNE2-EBV cells. One week after tumor inoculation, mice were treated with mRNA vaccine and NK cells (Vac+NK), NK cells alone (NK), or liposomes and PBS (Control) every 3 to 4 days (*n* = 6 per group). Blood and tumor samples were collected for analysis at the end of the experiment. (B) Tumor growth curves of each group (ordinary two-way ANOVA with Tukey’s multiple comparisons test, ∗∗∗*p* < 0.001, ∗∗∗∗*p* < 0.0001). (C) Photograph of harvested tumor tissues from each group. The cross symbol indicates eradicated tumors by the combined therapy. (D) Average tumor weight of each group (ordinary one-way ANOVA with Tukey’s multiple comparisons test, ∗*p* < 0.05, ∗∗∗∗*p* < 0.0001). (E) HE staining and (F) IHC staining of EBNA1 in tumor sections from each group. Scale bars: 500 μm (top), 100 μm (bottom, enlarged boxed regions). (G) Body weight and food intake changes of PBMC-humanized mice in the control, NK, and Vac+NK groups at different time points (*n* = 6 per group). A group of non-humanized NOG mice was additionally recorded for reference. (H) Tumor growth curves of each group from humanized mice reconstituted with PBMC source from donor #3 (ordinary two-way ANOVA with Tukey’s multiple comparisons test, ∗∗*p* < 0.01, ∗∗∗∗*p* < 0.0001). All data represented as means ± SEM.

After the abovementioned safety profile assessment, we next asked whether combining NK cells with mRNA vaccine could lead to improved therapeutic efficacy against EBV^+^ NPC. We evaluated the tumor control ability of the combined therapy by multiple vaccination of nEL vaccine and intravenous injection of allogenic NK cells in the humanized mouse EBV^+^ NPC model (Figure 5A). Compared to the control group treated with PBS containing 1% human serum albumin intravenously and liposomes intramuscularly, both combined therapy and NK cell therapy alone resulted in significant tumor growth inhibition (Figure 5B). Notably, the combined therapy exhibited a stronger anti-tumor effect and the ability to effectively eradicate established NPC in humanized mice. Complete tumor regression was observed in 50% of the mice (three out of six) treated with the combined therapy (Figure 5C). Furthermore, the average weight of remaining tumors from the group treated by the combined therapy was also significantly lower than that of other groups (Figure 5D).

To further characterize the tumor-reduction effect of the combined therapy, we performed H&E staining in tumor sections obtained from all groups. Tumor burden in mice treated with the combined therapy was lower compared to control or NK-cell-treated mice as shown by histological image analysis (Figure 5E, upper panel). The magnified views of tumor sections from both NK-cell and combined-therapy-treated mice revealed the presence of loose cancerous tissues resembling liquefactive necrosis, accompanied by a massive infiltration of immune cells (Figure 5E, lower panel). In parallel, we also examined changes of EBV oncogene expression in NPC after treatment. The histological analysis of tumor sections immunostained with a specific anti-EBNA1 antibody demonstrated the ubiquitous expression of this oncogene in control mice. In contrast, substantial regions of tumors treated with either NK cells or combined therapy showed significantly diminished levels of EBNA1 in parts of necrosis, suggesting reduced EBNA1 replication or elimination of tumor cells following treatment (Figure 5F). During the observation period, the combined therapy was well tolerated and exhibited a robust safety profile, as evidenced by the absence of body weight loss or abnormal food intake behavior in all treated mice (Figure 5G).

Regarding the generalizability of our combination therapy across heterogeneous genetic backgrounds, we implemented rigorous validation, which utilized humanized NOG mice reconstituted with PBMCs from another pre-screened donor with an HLA type (donor #3, Table S1) that is substantially different from the previous donor (donor #1, Table S1). Experimental data demonstrated that the combination therapy can still effectively inhibit EBV^+^-NPC tumor progression in humanized-mice with a different genetic background (Figures 5H and S4). Quantitative analysis revealed comparable tumor suppression efficacy to initial results obtained from donor #1, with 7.1% inter-donor variation in therapeutic response (Table S2). This multi-donor validation confirms the robustness of our strategy across distinct human PBMC sources.

### Characterizing the underlying immune response elicited by the combined therapy

**Figure 6:**
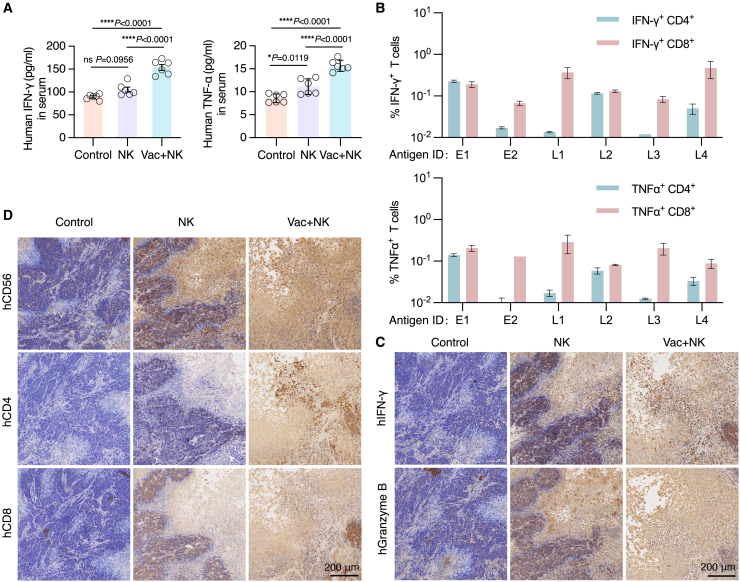
The combined therapy mobilizes both T cell and NK cell immunity against EBV^+^ NPC in humanized mice (A) Quantification of the serum concentrations of human IFN-γ (left) and human TNF-α (right) in humanized mice from each group (ordinary one-way ANOVA with Tukey’s multiple comparisons test; ns, no significance; *p* > 0.05, ∗*p* < 0.05, ∗∗∗∗*p* < 0.0001; data are represented as means ± SEM). (B) Characterization of T cell subtypes induced by individual predicted EBV antigen by intracellular cytokine staining. Data (means ± SEM) show the percentages of IFN-γ^+^/TNF-α^+^ CD4^+^ and IFN-γ^+^/TNF-α^+^ CD8^+^ T cells and are representative of two experiments. (C) IHC staining of human IFN-γ and granzyme B in tumor sections from each group. (D) IHC staining of human CD56, CD4, and CD8 in tumor sections from each group. Scale bar, 200 μm.

To gain further insights into the underlying immune response driving the observed therapeutic effect, we collected serum samples from all mice and detected higher levels of systemic human tumor necrosis factor alpha (hTNF-α) and IFN-γ (hIFN-γ) in combined-therapy-treated mice when compared to control and NK-cell-treated mice (Figure 6A). This implies a synergistic induction of enhanced anti-tumor response through the combined therapy. We then analyzed the antigen-specific CD4^+^ or CD8^+^ responses elicited by individual epitope of the mRNA vaccine for combined-therapy-treated humanized mouse reconstituted with PBMCs from donor #3. We determined the subtype T cell responses by intracellular cytokine staining (ICS) and fluorescence-activated cell sorting (FACS). Among the six EBV antigens used, three of them (E2, L1, and L3) induced CD8^+^ T cell responses and the other three (E1, L2 and L4) were able to induce both CD8^+^ and CD4^+^ T cell responses (Figures 6B and S5), showing the induction of a well-balanced adaptive immune response.

Considering the extensive infiltration of immune cells observed in H&E-stained tumor sections from treated mice (Figure 5E), we further investigated the immune response evoked within their TME by immunohistochemistry (IHC) staining. As shown in Figure 6C, the levels of effector cytokines, human granzyme B and IFN-γ, were found to be significantly elevated in NK-cell- and combined-therapy-treated tumors, suggesting the induction of strong cytotoxic effects in the TME. We also observed intensive human NK cell infiltration in above treated tumors by staining human CD56 (Figure 6D, upper panel). Further analysis of the T-cell-mediated response through immunostaining of human CD4 and CD8 revealed significantly elevated levels of T cells, particularly infiltrating CD4^+^ T cells, in tumors treated with the combined therapy, as compared to those treated with NK cells alone or control tumors (Figure 6D, middle and lower panel). Collectively, the abovementioned results indicate that the combined mRNA vaccine and NK cell therapy can simultaneously elicit potent T-cell- and NK-cell-mediated immune responses against EBV^+^ NPC in humanized mice.

## Discussion

Despite the invaluable contribution of traditional mouse tumor models in cancer research, their ability to enable comprehensive *in vivo* evaluation of human-specific immunotherapies remains challenging.^53^ Humanized mice transplanted with both human immune cells and tumors simultaneously have currently emerged as promising preclinical models in immuno-oncology research. In previous studies, a hematopoietic stem cell (HSC)-humanized NSG mouse model with EBV^+^ NPC xenografts was established to evaluate combination therapy of the immune checkpoint inhibitors nivolumab and ipilimumab.^54^ HSC-humanized mice with spontaneous EBV^+^ lymphomas were also established for the evaluation of EBV-specific T cells.^55^ In this work, we used PBMC-humanized mice implanted with undifferentiated NPC to assess the effectiveness of mRNA vaccines as well as the concurrent therapy. Although PBMC-humanized NOG mice exhibit limited capacity to elicit antigen-specific humoral response, they enable the restoration of human cellular immunity, making them particularly useful for investigating human immune responses to cancer vaccines and adoptive cell therapies.^50^ Indeed, an adenovirus-based HPV vaccine has shown promising results when tested in PBMC-humanized mice harboring HPV16^+^ human cervical tumors by inhibiting tumor growth and enhancing T cell infiltration in the TME.^56^ A key limitation of PBMC-humanized NOG mice is the rapid development of GvHD, making long-term efficacy studies difficult.^53^ In this study, we pre-screened donor PBMCs to ensure compatibility with the murine hosts and NPC tumor model, which helps minimize the impact of GvHD on the experimental readout and extend the therapeutic window.

The induction of T cell responses typically relies on the recognition and HLA restriction of epitope sequences. Most EBV-related cancer vaccines in preclinical and clinical studies have been developed using viral epitopes derived from the standard EBV genome. It is now widely accepted that amino acid mutations in viral proteins can lead to altered systemic immune effects.^57^ To search for epitopes directly from cancer-associated EBV strains that undergo extensive mutations,^29^^,^^30^ we analyzed non-synonymous mutations in the viral genomes. High-frequency nsSNVs leading to single amino acid changes were discovered in EBV oncoproteins (Figure 1B). To develop a vaccine with maximum population coverage, we predicted common epitopes from these mutations with both strong HLA binding affinity and broad HLA coverage. A total of six mutation-encoding epitopes were selected for mRNA vaccine construction (Table 1). In the immunogenicity assay of the vaccine, T cell responses against each of the epitopes were successfully detected in humanized mice transplanted with PBMCs from two healthy donors with different HLA types (Figures 2E and 6B), suggesting that the epitopes may not only be restricted by specific HLA molecules. However, further experimental validation is needed to determine whether those epitopes can be presented by a wide spectrum of HLA alleles.

Although our mRNA vaccine could elicit antigen-specific T cell responses, therapeutic vaccination alone could not achieve a strong tumor-inhibiting effect. The suboptimal effectiveness of the vaccine can be attributed to several reasons. First, optimizing mRNA stability and translational capacity (e.g., through pseudouridine modification), timing and dosage of the vaccination, as well as vaccination routes may be critical to further improve vaccine efficacy.^58^ Second, the reconstituted immune system of PBMC-humanized mice comprises a limited population of human antigen-presenting cells (APCs), such as dendritic cells.^53^ The inadequate antigen presentation due to the lack of APCs may also affect the effectiveness of the vaccine. Although EBV^+^ NPC is a typical immune hot tumor with densely infiltrated immune cells, previous studies have shown that these cells are often dysfunctional, exhausted, and upregulated with inhibitory immune checkpoints.^59^^,^^60^ Therefore, the established immunosuppressive TME and other immune cell evasion mechanisms are likely to impair the anti-tumor response elicited by vaccination.

Combining cancer vaccines with therapies that target the immune-suppressive TME or reinvigorate tumor-specific T cells holds promise for maximizing the clinical benefit of vaccination. Due to their potent anti-tumor activity and favorable safety profile in allogeneic infusion, NK cells also represent an attractive but often overlooked co-treatment option. One study showed that a multi-modal therapy combining a neuroblastoma vaccine, NK cells, and checkpoint blockade, significantly suppressed mouse neuroblastoma after bone marrow transplantation.^61^ In our study, we utilized allogeneic NK cells for adoptive infusion. The decision to employ allogeneic instead of autologous NK cells was driven by both practical and biological considerations rooted in the limitations of autologous approaches. First, large quantities of NK cells are required for treatment in our experiments. The starting materials namely the donor PMBCs are limited for the expansion of enough autologous NK cells to fulfill the experimental requirement. In practical, the “off-the-shelf” availability of allogeneic NK cells also enables rapid deployment alongside tumor vaccines, a feature critical for treating aggressive cancers requiring immediate intervention. Second, while autologous NK cells are inherently HLA-matched and theoretically reduce graft rejection risks, their utility is often compromised by functional exhaustion, tumor-induced immunosuppression (e.g., downregulated NKG2D),^62^ and prolonged manufacturing timelines, particularly in patients with advanced malignancies. In contrast, allogeneic NK cells derived from healthy donors exhibit superior baseline cytotoxicity and intrinsic resistance to TME-driven suppression, thereby enhancing tumor targeting.^63^ Preclinical data further demonstrate that allogeneic NK cells synergize robustly with tumor vaccines through direct tumor lysis, releasing antigenic debris to amplify vaccine-primed CD8^+^ T cell responses,^43^ while concurrent depletion of immunosuppressive myeloid-derived suppressor cells (MDSCs) and regulatory T cells (Tregs) converts immunologically “cold” tumors into “hot” niches characterized by elevated CD8^+^ T cell infiltration and IFN-γ secretion.^64^^,^^65^ This approach harnesses the functional resilience and enhanced efficacy of allogeneic NK cells to address the limitations of autologous therapies while amplifying combinatorial antitumor immunity.

We found that concurrent treatment with the EBV mRNA vaccine and allogenic NK cells demonstrated greater tumor control or eradication capability than either monotherapy (Figure 5). This observation warrants further investigation into the underlying mechanism(s) of the synergistic effect. We exclude the plausibility of donor alloreactive T-cell-mediated tumor rejection, as the alloreactivity of donor T cells is the major cause of GvHD and plenty of safety profile data have demonstrated negligible GvHD by PBMC infusion or any form of treatment within the experiment window (Figures 4, 5G, S1, and S3). We clearly showed that the mRNA vaccine elicited significant epitope-specific T cell responses (Figures 2E and 6B). These responses were specific to the predicted epitopes and thus were not likely due to alloreactive T cells. Additionally, previous studies have shown that NK cells can actually suppress alloreactive T cell responses.^66^ This further strengthens the argument that the observed therapeutic efficacy is due to the synergistic action of the mRNA vaccine and NK cells, rather than alloreactive T cell activation. NPC cells exhibit heterogeneity in the expression of HLA molecules, with a significant proportion of cells having low or no HLA expression.^67^ These cells tend to evade the immune surveillance of cytotoxic T cells, but they can be recognized and eliminated by NK cells since HLA loss can serve as a “missing self” signal to trigger NK-cell-mediated cytotoxicity. Therefore, our concurrent therapy can exert multilayered anti-tumor effects to overcome the heterogeneity of NPC, as evidenced by the dense infiltration of both human T cells and NK cells in the TME (Figure 6D). Furthermore, there may be a complex interaction between NK cells and antitumor T cells, which may further enhance the synergistic effect.^68^ A previous study found that NK-cell-mediated tumor killing via CD27-CD70 interaction can facilitate the development of protective anti-tumor T cell immunity.^69^ Other studies have shown that NK cells may provide an early source of IFN-γ, which is necessary for polarization of Th1 cells,^70^ and Th1 cells will cooperatively promote the proliferation, activation, and infiltration of CD8^+^ cytotoxic T cells for tumor eradication.^71^

In summary, we exploited that combining EBV mRNA vaccine and adoptive NK cells constituted a novel strategy for the treatment of EBV^+^ NPC in humanized mice. By eliciting both adaptive and innate anti-tumor immune responses, our concurrent therapy holds the potential to overcome immune evasion and exert effective tumor control. Further studies are required to fully elucidate the synergistic mechanisms and long-term benefits of this combined strategy in the treatment of EBV^+^ NPC.

## Materials and methods

### EBV epitope prediction

EBV genome sequencing data were collected from BioProjects (PRJNA522388 and PRJEB24495),^29^^,^^30^ and sequence reads were mapped to EBV reference genome (RefSeq: NC_007605) by BWA-mem.^72^ Genomic variants were called with the help of HaplotypeCaller.^73^ After variant quality filtration, mutated peptides were submitted for binding affinity prediction for different classes of HLA alleles by NetMHCpan-4.1and NetMHCIIpan-4.0, respectively.^47^^,^^74^^,^^75^ Population coverage was calculated based on the HLA alleles with strong binding affinity to each predicted EBV epitope.^76^

### mRNA vaccine construction and administration

A plasmid template, designated as pRV1, was developed for mRNA synthesis based on a DNA backbone we previously reported.^77^ In brief, xenopus β-globin 5′ UTR and VEEF 3′ UTR were inserted to the pUC57 plasmid, and a copy of 130 bp poly-adenine (A) tail was cloned behind the 3′ UTR. The nEL, which stands for linked ENBA1, LMP1, LMP2A, and LMP2B epitopes with (GS)_5_ as a linker, or EGFP served as coding sequences to be subcloned into pRV1. Each epitope is encoded as a 30-mer amino acid peptide with mutated amino acid at position 15. Both coding sequences were fused in frame with an Igk signal peptide at the 5′ end and an MHCI trafficking domain (MITD) with a 6×His-tag at the 3′ end to yield pRV1-nEL and pRV1-EGFP. The plasmids were linearized by SapI digestion and *in vitro* transcribed using the HiScribe T7 mRNA Kit (NEB, E2040). mRNA products were purified by lithium chloride precipitation and 5′-capped using the vaccinia-virus-capping system (NEB, M2080). Capped mRNA products were stored at −80°C until usage. mRNA vaccines were formulated by mixing nEL mRNA with InstantFECT liposomes donated by PGR-Solutions^78^ and vaccinated every 3–4 days for a total of three treatments. For each therapeutic vaccination, 10 μg of nEL mRNA diluted with serum-free medium were mixed with 20 μL of 0.9% NaCl and 4 μL of InstantFECT to a total volume of 100 μL. The mixture was intramuscularly injected on the right and left flank of the mouse, with 50 μL administered to each site.

### Cell lines and cell culture

K562 and HEK-293T cell lines were purchased from ATCC. The EBV^+^ human NPC cell line CNE2-EBV was kindly provided by Prof. Xin-yuan Guan from the University of Hong Kong. All cell lines were authenticated with Material Transfer Agreement and tested for mycoplasma contamination. All cells were maintained in RPMI 1640 medium (Gibco, 11875093) supplemented with 10% FBS (Gibco, 10099141), 100 U/mL penicillin, and 100 mg/mL streptomycin (Gibco, 15140122) at 37°C in 5% CO_2_-humidified incubators.

### Humanized mice models

NOD.Cg-*Prkdc*^*scid*^ *Il2rg*^*tm1Sug*^/JicCrl (NOG) mice were purchased from Beijing Vital River Laboratory. All animal experiments were conducted in Specific Pathogen Free (SPF)-class housing of laboratory of Shenzhen LingFu TopBiotech Co., Ltd., under the guidance of Institutional Animal Care and Use Committee with granted No. TOP-IACUC-2023-0267. To establish humanized mice, human PBMCs were purified from buffy coats of healthy donors using Ficoll-Hypaque (Biochrom, L6115) density gradient centrifugation. Informed consents were obtained from PBMC donors for research approved by The Institutional Review Board of BGI with granted No. BGI-IRB 22046-T1. Donors were categorized based on HLA typing (A, B, C, DRB1, DQB1 loci). To prolong the humanized window while minimizing alloreactivity, PBMC engraftment efficiency (human CD45^+^ cell reconstitution), GvHD severity, as well as EBV^+^ NPC tumor engraftment efficiency were compared across multiple donors in pilot studies. Donor PBMCs showing <10% weight loss, delayed GvHD onset (>4 weeks post-transplantation), and successful EBV^+^ NPC tumor establishment are preferred. Isolated PBMCs from donors with pre-screened low alloreactivity were intravenously injected with an amount of 6×10^6^ per mouse. To establish a humanized-mouse EBV^+^ NPC model, two donors with HLA typing of A∗11:01 or A∗68:02 were selected for PBMC transplantation. Then, 5×10^6^ CNE2-EBV cells with HLA-A∗11 and A∗68 were injected subcutaneously into the right flank of the mice. Tumors were grown to an average volume of 50–100 mm^3^ before treatment. Tumor volume was calculated by measuring the length and width of each tumor using calipers, where V = length×width^2^×0.5. The GvHD pathogenesis (including weight loss, gastrointestinal distress, erythema, kyphotic posture, and pilomotor reflex abnormalities) is closely monitored to determine humane endpoints based on institutional animal welfare protocols to avoid inaccurate data collection due to alloreactivity.

### *In vitro* NK cell expansion and allogeneic NK cell infusion

PBMCs obtained from healthy donors were prepared as a source of NK cells. NK cells were amplified using our optimized protocol. Briefly, flasks were pre-coated with 5 μg/mL CD16 antibody (T&L Biotechnology, TL-201) and 25 μg/mL RetroNectin (Takara, T110A). Then PBMCs were seeded into coated flasks at 1×10^6^ cells/mL with 50 mL Start Medium and incubated at 37°C with 5% CO_2_. Start Medium was composed of X-VIVO 10 serum-free medium (Lonza, 04-380Q) supplemented with 1,000 IU/mL human IL-2 (Beijing Sihuan, S20040008), 10 ng/mL human IL-15 (Med Chem Express, HY-P7371), 0.1 μmol/mL GSK3βi-TWS119 (Med Chem Express, HY-10590), and 10% plasma substitute (Jiake, X990206). Refreshing Start Medium was performed at day 3. To initiate NK cell expansion, NK cells was transferred into a gas permeable culture bag (Nipro, NCB-22) after 7 days, and expanded cell populations were fed with Maintenance Medium for up to 21 days. Maintenance Medium was composed of X-VIVO 10 serum-free medium supplemented with 1,000 IU/mL human IL-2 and 1% plasma substitute. AO/PI (Countstar, 1250T) staining was used to calculate the total cell number and cell viability. The purity of NK cells was calculated by quantifying CD56/CD16^+^ CD3^−^ NK cells on day 21. For adoptive NK cell therapy, NK cells expanded separately from two donors were counted and resuspended in saline solution containing 1% human serum albumin (HuaLan Bio, S10950009); 1.5×10^7^ NK cells were intravenously administered to mice for a total of three treatments.

### Flow cytometry

NK cells were identified by surface staining of anti-human CD3 (BD, 552852), CD56 (BD, 555518), and CD16 (BD, 561304) antibodies. For the phenotypic analysis of NK cell activation, anti-human NKG2D (BD, 561815) and NKp30 (BD, 558407) antibodies were used to stain expanded NK cells. For the analysis of the effector function of NK cells, NK cells were pre-incubated with Brefeldin A (BD, 555029) for 5 h and then permeabilized and fixed using BD Cytofix/Cytoperm (BD, 554722) following manufacturer’s instructions. After permeabilization, intracellular cytokine staining was performed by using anti-human granzyme B (BD, 561142) and perforin (BD, 567722) antibodies. For subtyping antigen-specific CD4^+^ or CD8^+^ T cell responses, splenocytes of the treated mice were stimulated with each synthetic antigen peptide at a final concentration of 5 μg/mL in the presence of Brefeldin A. After overnight stimulation, surface staining of CD4 (BD, 551162) and CD8 (BD, 566852) and intracellular staining of TNF-α (BD, 563418) and IFN-γ (BD, 551385) were performed following manufacturer’s instructions. Samples were acquired on NovoCyte Advanteon or Beckman CytoFLEX Flow Cytometer Systems.

### Analysis of NK cell cytotoxicity

NK cells were co-cultured with 1 × 10^4^ K562 or 1×10^4^ CNE2-EBV as target cells at varying effector/target ratios (5:1, 10:1, 20:1, and 50:1). Effector/target cell mixtures were spun down in 96-well plates at 100 g for 2 min and then incubated at 37 °C for 4–6 h. Culture media were subsequently analyzed with LDH Cytotoxicity Assay Kit (Beyotime, C0017). Percentage (%) of NK specific killing = 100× (OD of apoptotic cells in co-cultures−OD of target cells−OD of effectors)/(OD of apoptotic cells−OD of target cells).

### ELISA

Human IFN-γ and TNF-α in mice peripheral blood were quantified using enzyme-linked immunosorbent assay (ELISA) kits (MEIMIAN, 0033H1 and 0122H1) following manufacturer’s instructions. Briefly, 2-fold serially diluted mice sera were added into plate wells and incubated for 1 h at 37°C. Plates were washed six times with PBS containing 0.05% Tween 20 and incubated with HRP-conjugated goat anti-mouse antibodies for 1 h at 37°C. Color was developed by using TMB solution, and absorbance was measured using an ELISA reader at 450 nm. Samples of pre-immune mouse sera were used as negative controls.

### Human IFN-γ ELISpot assay

hIFN-γ-producing splenocytes from vaccinated or control mice were analyzed using a cytokine-specific enzyme-linked immunospot (ELISpot) kit (Mabtech, 3420-2HPW) following manufacturer’s instructions. Briefly, splenocytes were plated at a concentration of 1×10^5^ cells/well and stimulated with 2 μg/mL different synthetic peptide (GL Biochem Shanghai Ltd.) for 20 h at 37°C. Splenocytes without peptide stimulation were used as negative controls. Cells were washed and incubated with biotinylated anti-IFN-γ mAb for 1 h at 37°C, followed by streptavidin-HRP conjugation for another 1 h at 37°C. Plates were then washed, and IFN-γ spots were developed for 20 min with BCIP/NBT substrate. Spots were recorded and counted using an immunospot analyzer.

### Immunostaining and histology

Tumors and tissues were fixed in 4% PFA solution (Servicebio, G1101) for 24 h, followed by dehydration in graded series of ethanol at 4°C until paraffin embedding procedure (Leica, EG1160). Paraffin sections (10 mm) were dewaxed in xylene and rehydrated with graded series of ethanol and ddH_2_O. For H&E staining, sections were stained with Harris hematoxylin and 2% eosin (Servicebio, G1005). For immunostaining, antigen retrieval was performed and then blocked with 10% goat serum (Solarbio, SL038) for 1 h, followed by incubation with primary antibodies against CD56 (1:200, Affinity, DF7832), granzyme B (1:200, Affinity, AF0175), perforin (1:200, Affinity, DF6004), CD4 (1:100, Abcam, DF16080), CD8 (1:100, Abcam, ab17147), IFN-γ (1:100, Affinity, DF6045), and EBNA1 (1:100, Santa Cruz, 81581) at 4°C for overnight. Finally, sections were incubated with corresponding HRP-labeled secondary antibodies for 30 min at 37°C and visualized using DAB Chromogenic Kit (Servicebio, G1212), followed by counterstaining with hematoxylin (Servicebio, G1004). Images were taken with Nikon Eclipse-E100 light microscopy.

### Statistical analysis

Statistical significance was determined by GraphPad Prism 8.0 using ordinary two-way ANOVA, unpaired two-tailed t test, ordinary one-way ANOVA, or as otherwise stated in the figure legends. *p* values were denoted in figures in the following way: ns: not significant; ∗*p* < 0.05; ∗∗*p* < 0.01; ∗∗∗*p* < 0.001; ∗∗∗∗*p* < 0.0001.

## Data availability

Data generated by the authors are available upon request.

## Acknowledgments

This work was supported by the 10.13039/501100001809National Natural Science Foundation of China (Grant no. 82003260 and 32001040) and 2023 Hangzhou West Lake Pearl Project Leading Innovative Youth Team Project (Grant no. TD2023017). We thank Prof. Xin-yuan Guan and Dr. Shan-shan Li from The University of Hong Kong for sharing cell lines and their valuable advices. We thank Dr. Ting-ying Xia for creating the mouse cartoon images.

## Author contributions

K.H., N.Z., and J.D.H. conceived the study. K.H., X.J.L., and T.Y.X. performed the experiments. J.C.H. performed bioinformatic analysis. N.Z. and K.H. analyzed the data, prepared the figures, and wrote the manuscript. F.P.X. and J.D.H. revised the manuscript. All authors read and approved the final manuscript.

## Declaration of interests

The authors declare no competing interests.

## Supplemental information

Document S1. Figures S1–S5 and Tables S1 and S2

Document S2. Article plus supplemental information
