# Supplementary material for: Epstein-Barr virus mRNA vaccine synergizes with NK cells to enhance nasopharyngeal carcinoma eradication in humanized mice
Source: Mol Ther Oncol. 2025 Apr 24;33(2):200986. doi: 10.1016/j.omton.2025.200986 (PMC12127627; doi:10.1016/j.omton.2025.200986)
Supplement: Document S1. Figures S1–S5 and Tables S1 and S2 [file mmc1.pdf]

OMTON, Volume *33*

Supplemental information

Epstein-Barr virus mRNA vaccine synergizes with NK cells to enhance nasopharyngeal carcinoma eradication in humanized mice

Kun Huang, Xiao-jun Lin, Jing-chu Hu, Ting-ying Xia, Feng-ping Xu, Jian-dong Huang, and Nan Zhou

Supplemental figures

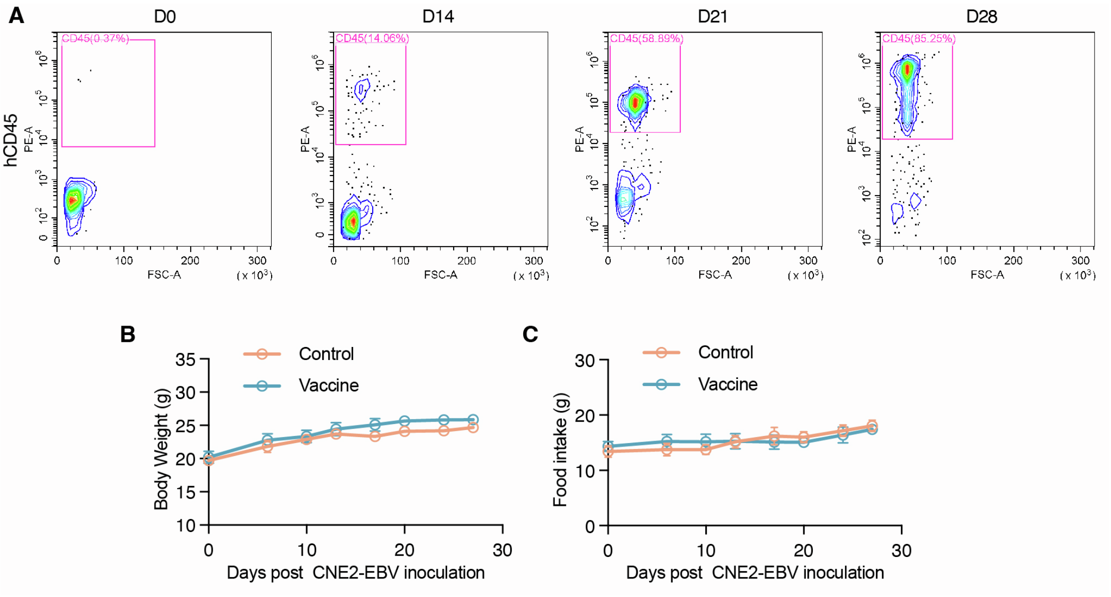

Figure S1. Establishment of human immunity in NOG mice for evaluating the immunogenicity and therapeutical potential of the mRNA vaccine. (A) Flow cytometric characterization of human CD45 expression in PMBCs from the humanized mouse at different time points post donor PBMC engraftment. (**B**) Body weight and (**C**) food intake changes of PBMC-humanized mice in the control (n=6) and vaccine group (n=5) at different time points. Data are presented as means ± SEM.

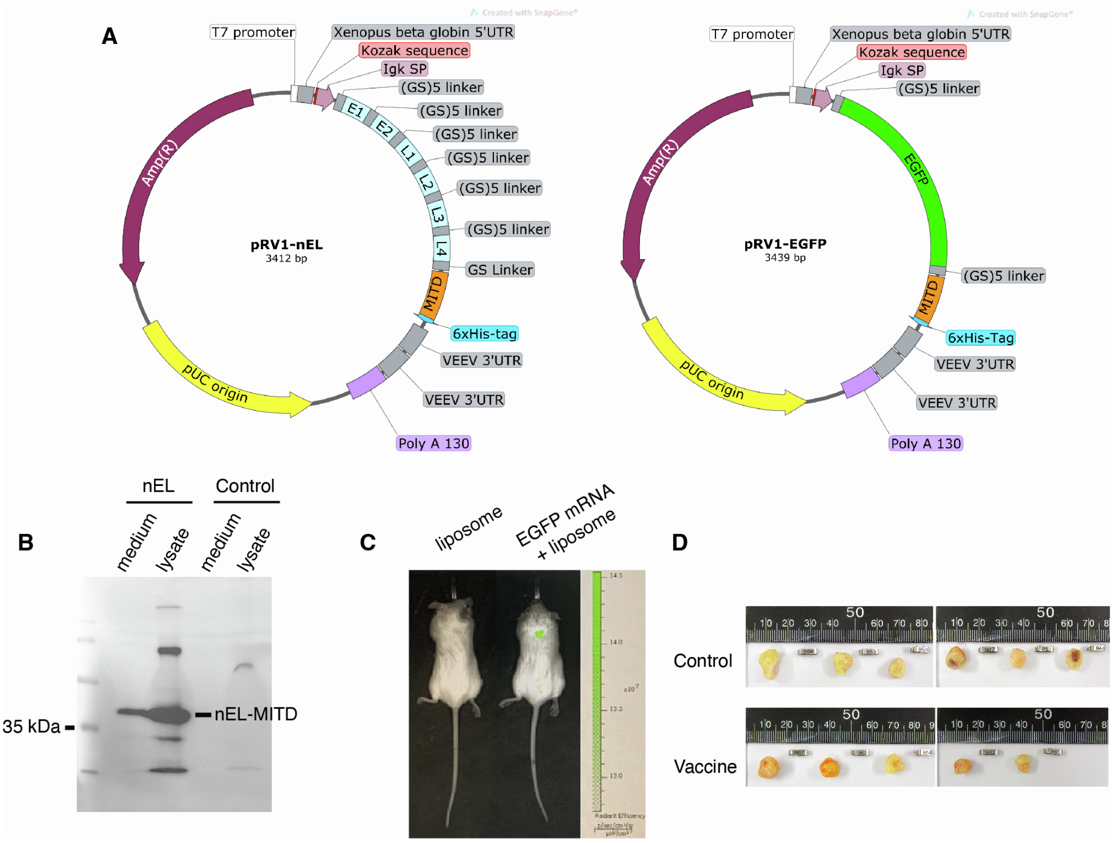

Figure S2. *In vitro* and *in vivo* expression of the designed mRNAs. (**A**) Plasmids maps for template plasmids used for producing nEL and EGFP mRNAs. Plasmid maps were created by SnapGene. (**B**) Immunoblots of culture medium and cell lysate of HEK-293T cells transfected with 500 ng of nEL mRNAs. Transfections were conducted with Lipofectamine MessengerMAX (ThermoFisher, LMRNA001). Samples obtained from untransfected cells were used as control. (**C**) 5 μg of EGFP mRNA complexed with 4 μl of InstantFECT liposomes were intramuscularly injected, and injection of liposomes alone served as control. *In vivo* imaging was performed for anaesthetized mice 24 h after injection. (**D**) Photograph of harvested tumor tissues from the control and vaccination group.

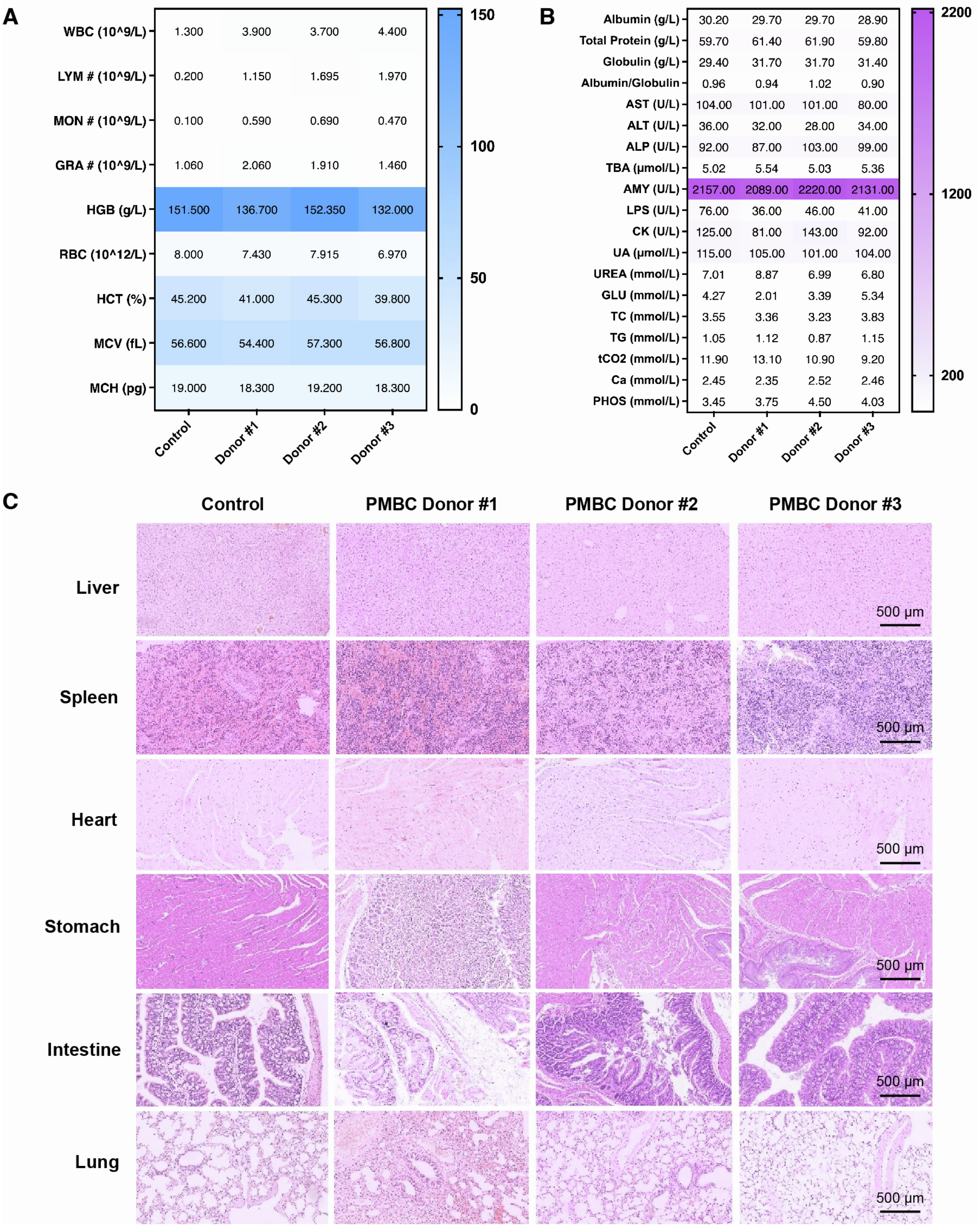

Figure S3. Safety evaluation of NOG mice transplanted with human PBMCs and allogenic NK cells. (**A**) Hematological parameters and (**B**) biochemical parameters of PBMC-humanized NOG mice compared to non-humanized NOG mice. Data are presented as medians in heatmaps. (**C**) HE staining for different organs of PBMC-humanized NOG mice compared to non-humanized NOG mice. Scale bars: 500 μm.

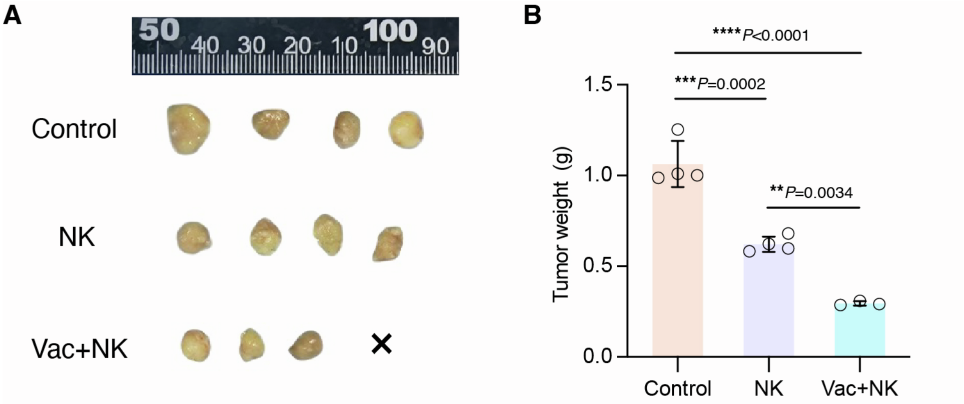

Figure S4. Efficacy of the combined therapy to promote the eradication of EBV^{+} NPC in humanized mice reconstituted from a different human PBMC source. (**A**) Photograph of harvested tumor tissues from each group. The cross symbol indicates eradicated tumors by the combined therapy. (**B**) Average tumor weight of each group (ordinary one-way ANOVA with Tukey's multiple comparisons test, $**P < 0.01$, $***P < 0.001$, $****P < 0.0001$). All data represented as means ± SEM.

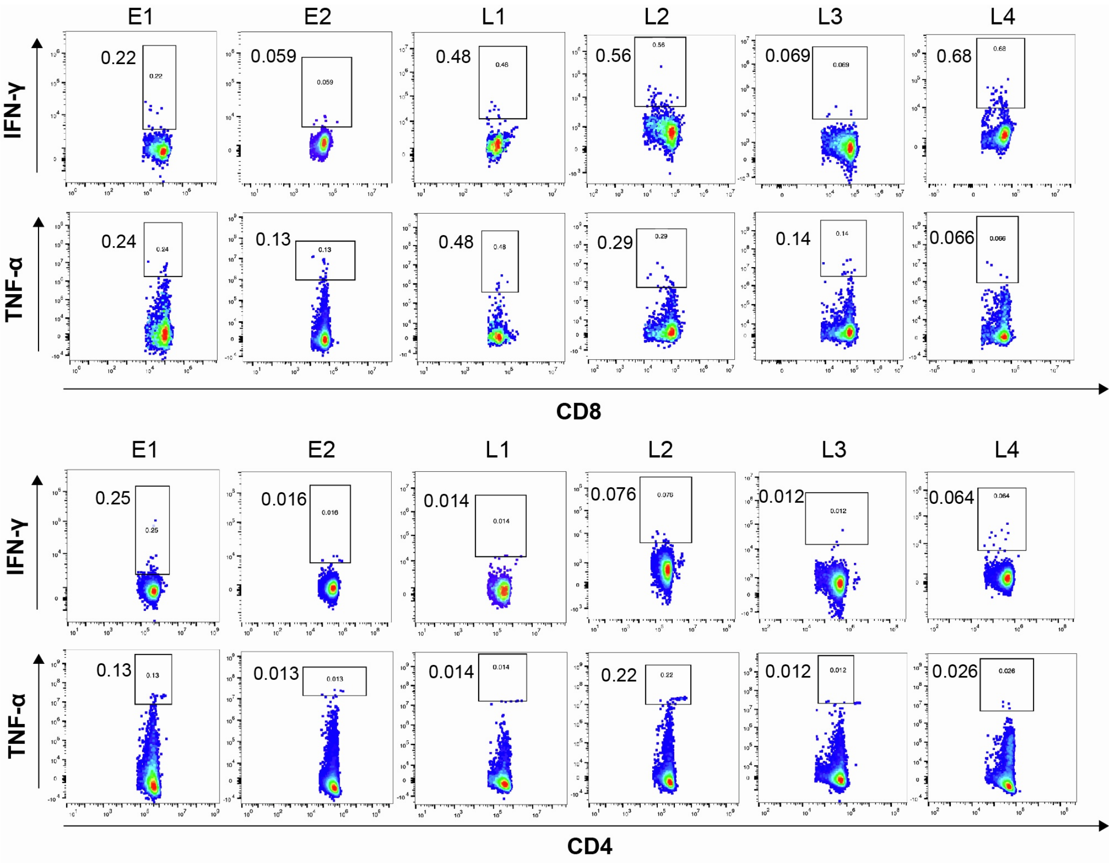

Figure S5. Detection and subtyping of antigen-specific T cells. Example flow cytometry IFN-γ and TNF-α responses stimulated by individual antigens. Spleenocytes from control mice stimulated with individual antigen peptide were gated as negative controls (% IFN-γ^+/ TNF-α^+ T-cell≤0.015) to set positive gating regions for antigen-specific T cell response in treatment groups.

Supplemental tables

Table S1. Donor characteristics and HLA genotyping. HLA alleles for classical loci (A, B, C, DRB1 and DQB1) are listed at high-resolution (two-field) typing for three healthy PBMC donors. PBMCs from donor #1 were used to develop humanized mice in Figure 2 and Figure 5B. PBMCs from donor #3 were used to develop humanized mice in Figure 5H.

**Donor**	**HLA genotyping**				
	A	B	C	DRB1	DQB1
#1	11:01,24:02	15:01,54:01	01:02,03:04	04:05,08:03	03:01,04:01
#2	31:01,69:01	51:02,52:01	12:02,15:02	07:01,09:01	03:03,03:03
#3	02:06,68:02	13:01,55:02	03:02,15:02	11:01,12:02	03:01,03:01

Table S2. Comparative quantitative assessment of antitumor efficacy in PBMC humanized mice from two distinct donor sources.

**PBMC source**	**Tumor rejection efficacy**		
	Control	NK	Vac+NK
Donor #1	0%	50.6%	76.5%
Donor #3	0%	31.3%	69.4%
